# Artificial Teeth Set in a Base of Block Tin

**Published:** 1850-10

**Authors:** 


*Artificial Teeth set in a Base of Block Tin.*—In another part of the present number of the Journal will be found two articles, one from Dr. E. G. Hawes, of New York, and the other from Dr. W. A. Royce, of Newburg, N. Y., on the use of tin as a base for artificial teeth. With regard to the value of tin for this purpose, we are unable to speak from experience. We saw a lower set secured in a base of this sort, which had a covering of gold, put on by the electro-galvanic process, constructed by Dr. E. G. Hawes, of New York. It certainly presented a most beautiful appearance, and if a coating of gold sufficiently thick can be put on to prevent it from wearing off and exposing the tin, and we would suppose it might be done, we know of no reason why a base of tin would not answer as good a purpose for artificial teeth for the lower jaw, as one composed altogether of gold. There are many cases where weight is desirable, and in such, it might be preferable. From the specimen put up by Dr. H., which we saw, we are disposed to think very favorably of it. It affords quite as secure an attachment for the teeth as the means usually employed for connecting them to a gold base. But time may be required to determine the real value of it.

